# Technical evaluation of a novel digital PCR platform for detecting *EGFR/KRAS* mutations in NSCLC archived plasma specimens

**DOI:** 10.1016/j.jlb.2023.100133

**Published:** 2023-12-19

**Authors:** Claudia Scimone, Francesco Pepe, Gianluca Russo, Lucia Palumbo, Glyn Ball, Pascale Morel, Alessandro Russo, Giancarlo Troncone, Umberto Malapelle

**Affiliations:** a Department of Public Health, University Federico II of Naples, Naples, Italy; b QIAGEN, Manchester, UK; c Department of Onco-Hematology, Papardo Hospital, Messina, Italy

**Keywords:** Digital-PCR, Liquid biopsy, NSCLC

## Abstract

**Introduction:**

*KRAS* p.G12C hot spot mutations has rapidly modified diagnostic algorithm for lung cancer patients electing Non-Small Cell Lung Cancer (NSCLC) patients to target treatment. As regards, patients that harbor this hallmark showed a clinical benefit in terms of progression-free survival (PFS) and overall survival (OS) in comparison with control group under target treatment. In this scenario, *KRAS* p.G12C mutation requires optimized testing strategy in diagnostic routine practice. Although the widespread diffusion of NGS platforms, a not negligible percentage of Italian diagnostic institutions adopt singleplex technology (RT-PCR, dPCR) for molecular testing. Here, we aim to technically validate a novel dPCR system (QIAcuity™ Digital PCR System, Qiagen; Hilden, Germany) on a retrospective series of cfDNA samples from previously tested with a custom NGS system. (1,2)

Methods: n = 50 liquid biopsy specimens (n = 25 *KRAS/EGFR* mutated and n = 25 wild type for actionable *KRAS/EGFR* mutations) from diagnostic routine NSCLC patients previously tested with a custom NGS panel were retrieved from our internal archival. Each sample was tested by adopting n = 5 *KRAS* and n = 3 EGFR commercially available dPCR assays on QIAcuity™ Digital PCR System (Qiagen; Hilden, Germany); an ultra-deep dPCR walk-away platform that automatizes molecular analysis. Technical sensitivity, technical specificity, and concordance rate between “gold standard” NGS system and QIAcuity™ Digital PCR System (Qiagen; Hilden, Germany) were assessed.

**Results:**

Overall, all specimens were successfully analyzed with dPCR system. In details, 24 out of 25 mutated and 21 out of 24 wild type cases were detected. A technical sensitivity, specificity, and a concordance rate of 96.0 % (24/25), 88.0 % (22/25) and 92.0 % (46/50) were evaluated taking into account a MAF cut-off ≥ 0.2 % and a partition number of 100 positive partitions in wild-type channel.

**Conclusion:**

Qiacuity (Qiagen; Hilden, Germany) platform enables accurate molecular analysis of diagnostic routine specimens. Optimized technical workflow is required to technically implement this platform in diagnostic routine setting.

## 1 Introduction

In the last decades, personalized medicine has revolutionized the clinical management of non-small cell lung cancer (NSCLC) patients [1,2]. Indeed, an ever-increasing number of therapeutic molecular biomarkers have been approved in clinical practice for the selection of NSCLC patients who would benefit from tumor-profile based treatments. Thus testing for these so called must-test genes is now mandatory and strongly recommended by the College of American Pathologists (CAP), the International Association for the Study of Lung Cancer (IASLC), and the Association for Molecular Pathology (AMP) [3]. The clinical use of predictive biomarkers has been coupled with the development and application of high-throughput sequencing technologies, not least among them ultra-deep next generation sequencing platforms (NGS). However, although NGS platforms have the unique advantage of simultaneously detecting relevant hot spot mutations in predictive biomarkers, a significant percentage of NSCLC patients do not undergo molecular testing because of insufficient tumor tissue material or total lack thereof [4,5]. This is a huge, missed opportunity for many of these patients if one considers that up to 90.0 % of NSCLC patients harbor Epidermal Growth Factor Receptor (*EGFR)* druggable mutations. In this scenario, liquid biopsy which entails a simple peripheral blood draw, constitutes a valid alternative source of nucleic acids (circulating free DNA) to evaluate *EGFR* mutations in everyday clinical practice. In particular, liquid biopsy is routinely used to detect clinically actionable variants within exons 18-19-21-21 EGFR sensitive mutations when tissue material is inadequate, thus helping to stratify NSCLC patients for first- or second-generation tyrosine kinase inhibitors (TKIs). Moreover, liquid biopsy is also a key diagnostic tool for the identification of acquired resistance mutations, namely, exon 20 p.T790 M, after NSCLC patients fail to respond to first or second line TKI treatments [[6], [7], [8]]. In addition to *EGFR*, other predictive biomarkers have been identified in NSCLC. Among them is Kirsten Rat Sarcoma Viral Oncogene Homolog (*KRAS*) exon 2 p.G12C mutation. Occurring in about 13.0 % of NSCLC patients, this mutation gained attention as a clinically actionable target after the FDA approval of the KRAS^G12C^ inhibitor sotorasib [9]. Similarly, V-Raf murine sarcoma viral oncogene homolog B (*BRAF*) gene mutations, detected in few cases of NSCLC patients, have emerged as predictive biomarkers in the clinical stratification of NSCLC patients who show clinical sensitivity to TKI treatments, particularly, vemurafenib and dabrafenib plus trametinib [10,11].

Despite the widespread diffusion of NGS platforms in referral centers, most predictive molecular pathology laboratories are unable to exploit the advantages of utilizing this technology mainly because of long turnaround time, shortage of high-skilled personnel, and batching limitations [12]. A potential solution to address these key challenges in everyday clinical practice would be to apply orthogonal technology to NSCLC liquid biopsy specimens in small-to medium-sized referral centers. Such approach enables clinical pathologists to identify and analyze molecular alterations with a mutation allele frequency (MAF) as low as 0.1–1.0 % [13]. In this scenario, digital PCR (dPCR) systems have proven to be highly sensitive to low abundance molecular alterations even when starting from scant input material [14]. Currently, a plethora of commercially available dPCR based assays are used for routine testing of actionable driver alterations in NSCLC patients [15]. The purpose of this study was to validate the technical sensitivity of a novel dPCR system namely, the QIAcuity digital PCR system (QIAcuity™ Digital PCR System, QIAGEN; Hilden, Germany) in detecting low frequency NSCLC mutations in liquid biopsy specimens. For this purpose, this dPCR system was applied to a retrospective series of cfDNA samples from diagnostic routine practice previously tested with a targeted NGS system.

## 2 Methods

### 2.1 Study design

**Fig. 1:**
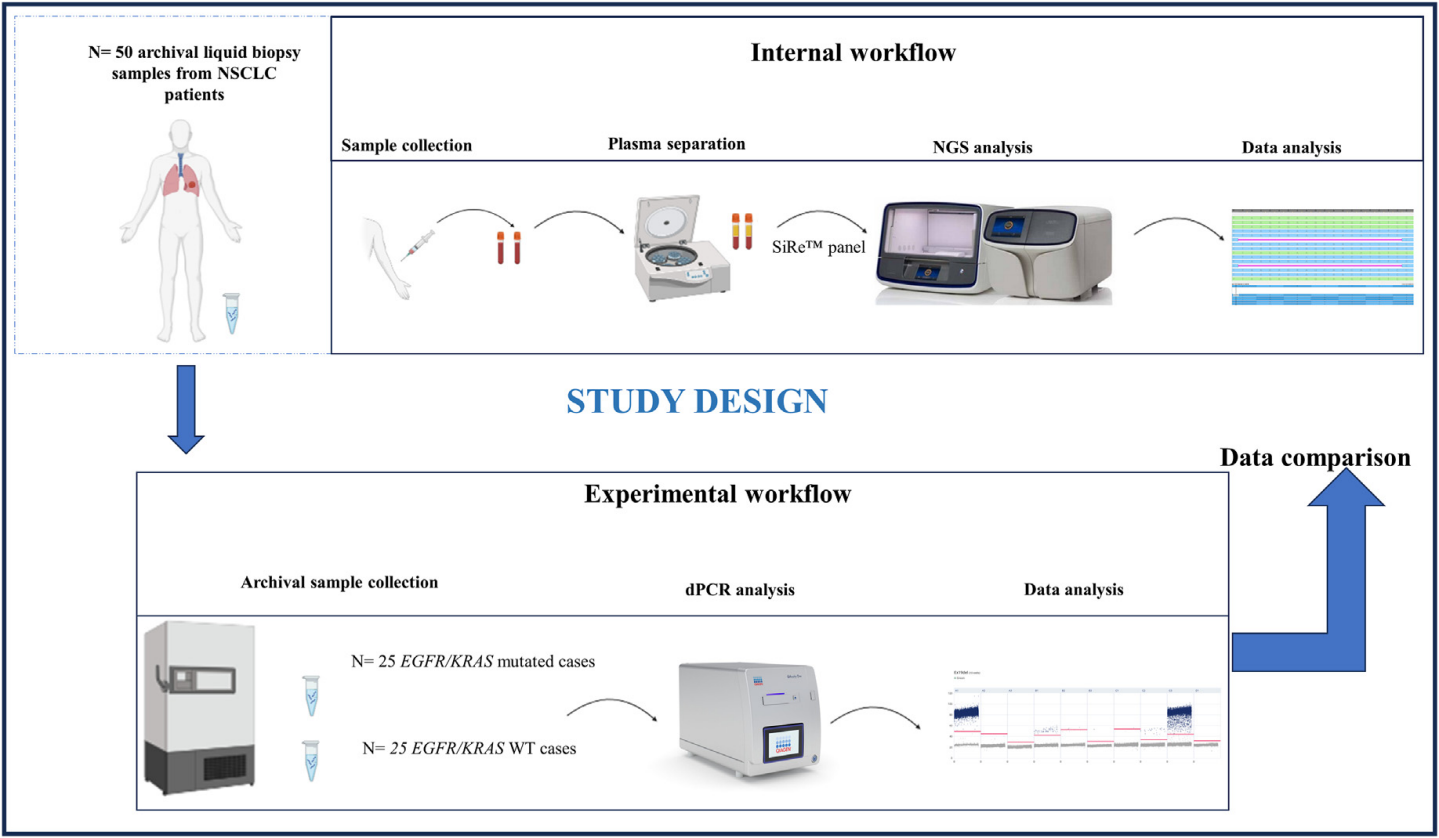
Schematic representation os stuty design.

Overall, we analyzed n = 50 liquid biopsy specimens from NSCLC patients previously tested for *KRAS/EGFR* with a custom NGS panel able to simultaneously detect 568 clinically relevant hot spot mutations in seven predictive biomarkers for solid tumor (SiRe™) [13]. Patient samples were retrieved from the internal archives of the predictive molecular pathology laboratory at the University of Naples Federico II. A series of cfDNA samples from n = 25 NSCLC patients positive for *KRAS/EGFR* and n = 25 cfDNA samples from NSCLC patients with no detectable *KRAS/EGFR* hotspot mutations were retrospectively selected (Table 1). Each sample was processed with n = 8 commercially available dPCR assays able to cover n = 5 *KRAS* and n = 3 EGFR clinically relevant hotspot alterations; in brief, each system comprised an ultra-deep dPCR walk-away platform able to integrate and automate the plate processing steps for the detection and measurement of low abundant MAF in liquid biopsy specimens [16]. Technical sensitivity, technical specificity, and concordance rate between “gold standard” NGS and QIAcuity™ Digital PCR were assessed. Written informed consent was obtained from all patients and documented according to The Italian Data Protection Authority (http://www.garanteprivacy.it/web/guest/home/docweb/-/docwebdisplay/export/2485392). All information regarding human material was managed with anonymous numerical codes, and all samples were handled in compliance with the Declaration of Helsinki (http://www.wma. net/en/30publications/10policies/b3/). (Fig. 1)

**Table 1:**
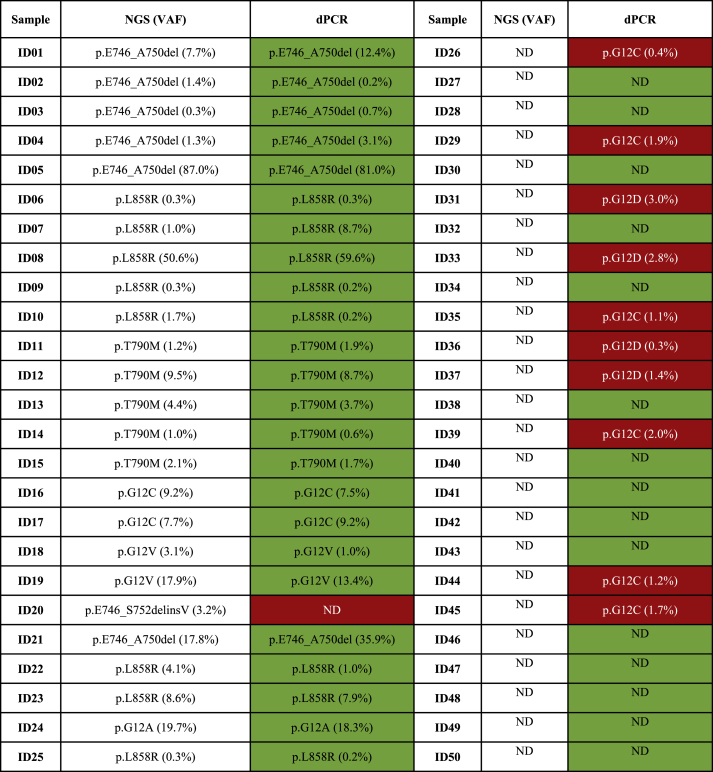
List of concordant (green) and discordant cases (red) evaluated by using QIAcuity system (Qiagen) in a retrospective series of n = 50 NSCLC patients previously tested with NGS custom panel.

Abbreviations: *dPCR* (Digital PCR); *NGS* (Next Generation Sequencing); *VAF* (Variant Allele Frequency); *ND* (Not detected).

### 2.2 Sample management

A total of 10 ml of peripheral blood was withdrawn from each patient and collected into K2 EDTA BD Vacutainer ® blood tubes (n = 2). Plasma was immediately separated from cell debris by double centrifugation at 2300 rpm for 10 min following the manufacturer's instructions. Finally, plasma specimens were aliquoted and stored at −80 °C until molecular analysis [13]. In particular cfDNA was automatically purified from plasma specimens with the QIAsymphony DSP Virus/Pathogen Midi Kit (QIAGEN, Hilden, Germany) [13]. Cell free circulating DNA was purified from 1.2 ml of plasma after processing. In the event that the plasma volume was inadequate (1.0 and 1.2 ml), PBS was added to obtain the standardized input volume recommended by the manufacturer [13]. Finally, the extracted cfDNA was eluted in 60 μl buffer and stored at −80 °C for further analysis.

### 2.3 NGS analysis

NGS library preparation was carried automatically. In brief, a series of n = 8 cfDNA specimens were simultaneously amplified and purified on the Ion Chef (Thermofisher). A total of 15 ng DNA was dispensed on Ion Code plates and amplified with the Ion AmpliSeq DL8 kit (ThermoFisher Scientific) by following standard thermal cycling conditions (23 cycles for the amplification step, 4 min each). Purified libraries were then diluted to 30 pM and loaded onto the Ion Chef instrument for template preparation. Finally, pooled libraries were automatically dispensed on Ion 520™ Chip and sequenced on Ion S5™ System (ThermoFisher Scientific) according to the manufacturer's instructions. Signal processing and base calling were set on the default base-caller parameters of Torrent Suite [v.5.0.2]. In particular, coverage analysis was conducted with customized SiRe™ designed bed files with coverage plug-in (v.5.0.2.0). Variant calling was carried out automatically by integrating the default variant caller plug-in (v.5.0.2.1) previously optimized for SiRe™ technical parameters and by visual inspection of the raw data on the Golden Helix Genome Browser v.2.0.7 (Bozeman,MT, USA). Molecular alterations were recorded only if they had an allele coverage ≥5X a quality score ≥20, within an amplicon that covered at least 1000X alleles, and a MAF level ≥ 0.2 %.

### 2.4 dPCR analysis

Overall, *EGFR/KRAS* molecular assessment was investigated with the QIAcuity™ Digital PCR System (QIAGEN; Hilden, Germany) according to the manufacturer's instructions. Briefly, 25 μl of purified cfDNA was combined with the QIAcuity Probe PCR Kit (QIAGEN, Hilden, Germany) and the commercially available primers and probes (dPCR LNA Mutation Assays; QIAGEN, Germantown, US), for each *KRAS/EGFR* mutated case. Similarly, 8 μl of archival cfDNA was used to cover all *KRAS/EGFR* target alterations successfully. Samples were manually loaded into the QIAcuity™ 26K 24-well Nanoplate (QIAGEN, Hilden, Germany) according to the manufacturer's instructions. Positive controls were synthetic gene fragments, e.g., gBlocks™ (Integrated DNA Technologies, Leuven, Belgium); each fragment was specific for the mutation and wild type sequences of each relevant molecular alteration in the *KRAS/EGFR* genes. Negative controls comprised reactions in which the sample diluent alone was added to the reaction. Amplification steps were carried out in accordance with the manufacturer**'**s thermal protocol. Partitioning, processing, and fluorescent signal processing was automatically carried out by proprietary software (QIAcuity® Software Suite Version 1.2). In brief, the total amount of target molecular alteration was calculated by multiplying the copies of DNA target per partition (calculated from the Poisson distribution) by the number of valid partitions in each well. The concentration was then calculated by referring to the partition volume.

## 3 Results

Overall, dPCR-based analysis of *EGFR/KRAS* in NSCLC was successfully carried out in all instances. The number of negative partitions, positive partitions, and copy numbers (ng/μl) evaluated in wild type and mutated fluorescent signals were the following: 24803.9 (ranging from 20123.0 to 25484.0) and 25186.8 (ranging from 17716.0 to 25496.0); 573.1 (ranging from 3.0 to 5280.0) and 188.0 (ranging from 0 to 7559.0); 28.7 (ranging from 0.1 to 283.7) and 10.5 (ranging from 0 to 432.6), respectively.

Moreover, comparable results were also observed in terms of negative partitions (25077.8, ranging from 24520.0 to 25466.0, and 24872.0, ranging from 17716.0 to 25479.0), positive partitions (96.8, ranging from 10.0 to 222.0, and 445.7, ranging from 0 to 7559.0), and copy number (ng/μl) (4.7, ranging from 0.5 to 11.0, and 24.9, ranging from 0 to 432.6 ng/μl) between *KRAS* and *EGFR* mutant fluorescent signals, respectively.

In addition, inspection of wild type fluorescent signals generated technically comparable results in terms of negative partitions (24260.4, ranging from 23143.0 to 25223.0, and 24556.0, ranging from 22276.0 to 25328.0), positive partitions (910.6, ranging from 213.0 to 1602.0, and 769.7, ranging from 137.0 to 3093.0), and copy number variations (45.3, ranging from 10.2 to 81.5, and 38.2, ranging from 6.6 to 158.3 ng/μl) in *KRAS* and *EGFR* based assays, respectively (Supplementary Table 1). In this regard, wild-type samples revealed 25111.0 negative partitions (ranging from 201230.0 to 25484.0), 348.4 positive partitions (ranging from 3.0 to 5280.0), and 17.8 copy number variations (ng/μl) (ranging from 0.1 to 283.7). (Supplementary Table 2).

Notably, matching molecular profiles from QIAcuity™ Digital PCR System (QIAGEN; Hilden, Germany) with those from archived NGS report were observed in 24 out of 25 (96.0 %) mutated cases and 15 out of 25 (60.0 %) cases with no detectable molecular alterations (Table 1). In this regard, a concordance rate of 78.0 %, a technical sensitivity of 96.0 %, and technical specificity of 60.0 % 96.0 % and 60.0 %, was reached.

Interestingly, as for samples with at least 100 partitions in the WT assay, 24 out of 25 (96.0 %) mutated and 21 out of 24 (87.5 %) wild type case cases were assessed. In addition, a concordance rate of 92.0 %, a technical sensitivity of 96.0 %, and specificity of 88.0 % were achieved. After a visual inspection of NGS raw data, low frequency mutations in patients ID#26, #36, and #37 were detected by the dPCR system. (Table 2), In this regard, an overall concordance agreement of 98.0 % (49 out of 50) was reached.

**Table 2:**
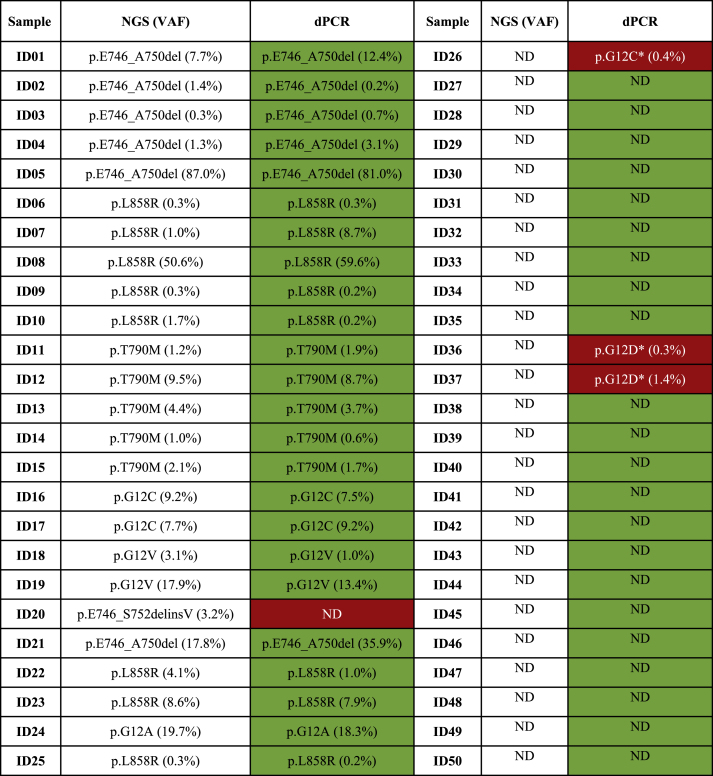
List of concordant (green) and discordant cases (red) evaluated by using QIAcuity system (Qiagen, Hilden, Hilden, Germany) in a retrospective series of n = 50 NSCLC patients previously tested with NGS custom panel. A cut off value of 100 positive partitions was set in wild-type channel.

Abbreviations: *dPCR* (Digital PCR); *NGS* (Next Generation Sequencing); *VAF* (Variant Allele Frequency); *ND* (Not detected).

## 4 Discussion

In today's routine diagnostic practice, the role of liquid biopsy in the clinical management of solid tumors is rapidly growing, thereby accelerating the need for new ultra-sensitive technologies [13,17]. Although NGS platforms have drastically impacted the diagnostic algorithm for molecular testing, their full use in small-to medium-sized institutions has been hampered by high costs, the need for and shortage of skilled personnel and slow turnaround time (TAT) [12]. These challenges have led to the development of alternative but equally sensitive technologies able to detect clinically actionable alterations even in scant diagnostic specimens. High reliability has been provided by digital PCR (dPCR) systems. In brief, these systems can identify low abundant molecular alterations (0.01–1.0 %) not detectable by conventional RT-PCR systems [14,15]. Particularly, dPCR application in clinical settings has been encouraged by the increasing implementation of liquid biopsy specimens to monitor minimal residual disease (MRD) and, equally important, to detect minor allele frequency (MAF) variants in driver biomarkers [18].

In this study, we verified the technical efficiency of a novel dPCR platform (QIAcuity™ Digital PCR System, QIAGEN; Hilden, Germany) by applying it to a retrospective series of archived NSCLC liquid biopsy specimens (n = 25 EGFR*/KRAS* wild type, n = 25 EGFR*/KRAS* mutated cases) previously tested with NGS. Comparative results between the two technologies were observed. An important advantage shown by QIAcuity dPCR was that its fully automated workflow allowed us to detect both mutations correctly in less turnaround time. Another advantage was that it enables us to carry out signal detection and data analysis using easily interpretable proprietary software, thereby increasing its technical sustainability in routine practice. Remarkably, QIAcuity dPCR also successfully analyzed each residual sample. Of note, similar technical parameters were observed when we compared *EGFR* and *KRAS*-based assays. We obtained a concordance rate of 78.0 % (39 out of 50), a technical sensitivity of 96.0 % (24 out of 25), and a specificity of 60.0 % (15 out of 25) by applying the default parameters generated by the proprietary software. Interestingly, we obtained a concordance rate of 92.0 (46 out of 50) a technical sensitivity of 96.0 (24 out of 25), and specificity of 88.0 % (22 out of 25) by setting a minimum technical cut-off value of 100 for the positive partitions in the wild-type channel. Of note, 14 out 25 (56.0 %) *EGFR/KRAS* wild type cases did not reach the technical cut-off threshold of 100 for the positive partitions in the wild-type channel.

**Fig. 2:**
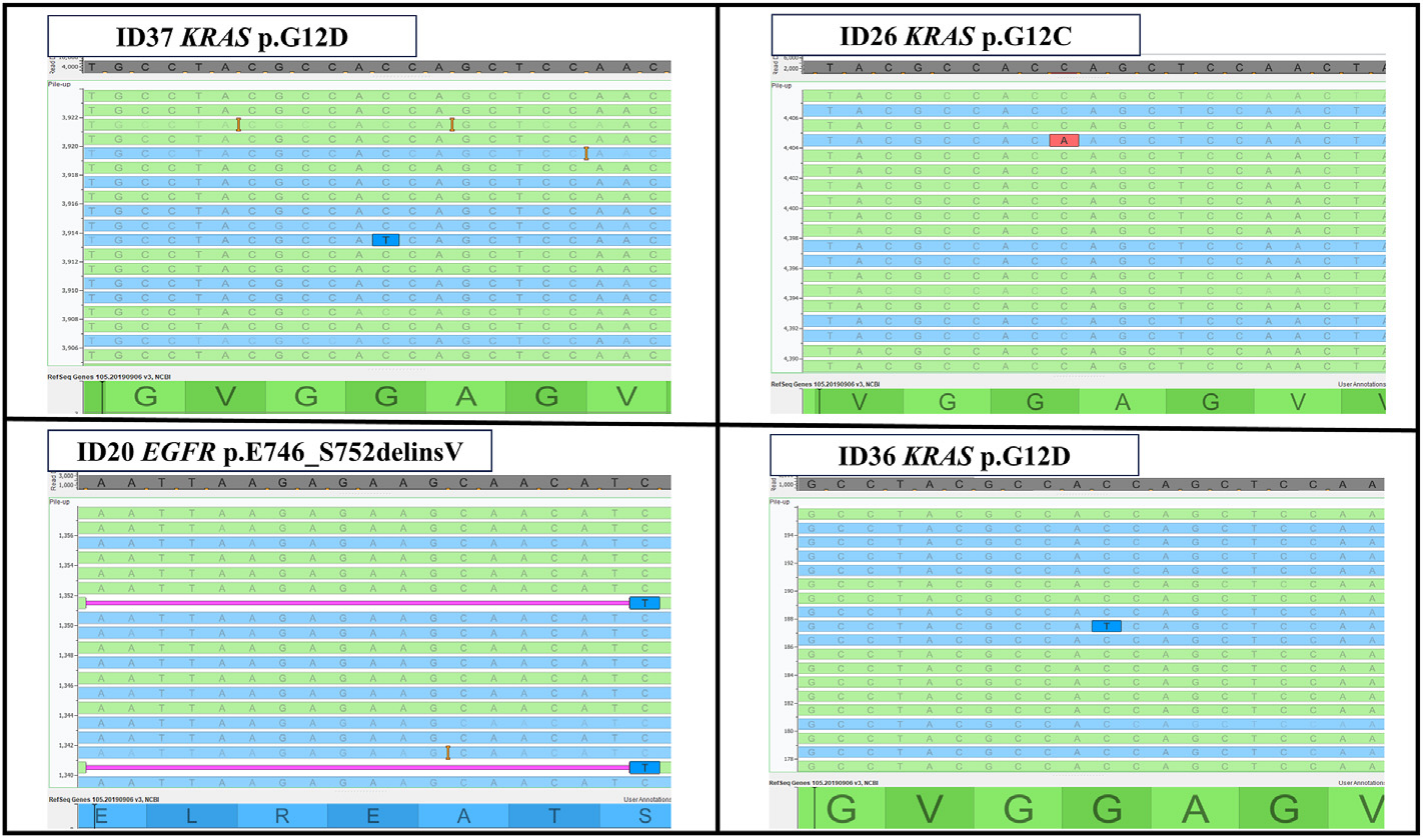
Visual inspection of raw data from NGS analysis confirmed *KRAS* p. G12D (ID 36, ID 37) and *KRAS* p.G12C (ID 16) hot spot mutations detected by Qiacuity (Qiagen, Hilden, Germany). Conversely, p.E746_S752delinsV EGFR mutation was only identified by NGS platform.

Our study also pointed out that the starting amount of nucleic acids is crucial to obtain successful molecular test results. In fact, by adopting 25 μl of mutated samples, we were able to satisfy this technical parameter, thereby improving the technical sensitivity and specificity of the assay. For instance, the QIAcuity assay was able to capture *KRAS* positive signals in three cases (ID#26 p.G12C, ID#36 and ID#37 p.G12D), whereas the NGS system missed them. Our visual inspection of NGS raw data confirmed the same molecular profile in all instances (Fig. 2). However, we did not include these molecular alterations in the clinical report because ID#26 had been tested before sotorasib was approved for p.G12C *KRAS* mutation-positive NSCLC patients. Conversely, p.G12D *KRAS* mutations were not informative for the clinical stratification of NSCLC patients. When applied to the NGS positive cases, the QIAcuity dPCR system detected different molecular profiles in one out of 25 (4.0 %) patients. Conversely, it missed the complex exon 19 c.2237_2255delinsT, p.delELREATSinsV *EGFR* molecular alteration previously detected with NGS. This happened because the probes selected for the analysis were not designed to cover this particular alteration. In the future, this problem could be easily solved by designing more target-specific probes [19,20].

Overall, QIAcuity dPCR proved to be as accurate as NGS in detecting even low frequency molecular alterations in NSCLC liquid biopsy samples. Indeed, we were able to assess low frequency *KRAS* mutations in all false positive cases, reaching an overall agreement of 98.0 % (49 out of 50 cases).

Although this study shows encouraging preliminary evidence for the implementation of QIAcuity™ Digital PCR System in routine clinical practice, it did have few limitations. One limitation was the retrospective nature of the study; the other was the small series of NSCLC liquid biopsy specimens analyzed, Furthermore, only *EGFR/KRAS* mutated cases were collected. Thus, further investigations are warranted to technically validate the partition cut-off values in a large prospective series of real-world cases covering all clinically druggable molecular alterations in NSCLC patients. Indeed, further investigations could provide better insights into the feasibility of integrating the QIAcuity™ Digital PCR System may be considered a technically reliable approach integrating in the routine diagnostic workflow of NSCLC liquid biopsy specimens.

## Author contributions

Conceptualization: CS, FP, UM; Data curation: all authors; Formal analysis: all authors; Funding acquisition: all authors; Investigation: all authors; Methodology: all authors; Project administration: all authors; Resources: all authors; Software: all authors; Supervision: all authors); Validation: all authors; Visualization: all authors; Roles/Writing - original draft: all authors; and Writing - review & editing: UM.

## Declaration of competing interest

The authors declare the following financial interests/personal relationships which may be considered as potential competing interests: Umberto Malapelle reports equipment, drugs, or supplies was provided by Qiagen UK. Glyn Ball reports a relationship with Qiagen UK that includes: employment. Pascale Morel reports a relationship with Qiagen UK that includes: employment. Giancarlo Troncone has received personal fees (as speaker bureau or advisor) from Roche, MSD, Pfizer, Boehringer Ingelheim, Eli Lilly, BMS, GSK, Menarini, AstraZeneca, Amgen and Bayer for work performed outside of the current study. Umberto Malapelle has received personal fees (as consultant and/or speaker bureau) from Boehringer Ingelheim, Roche, MSD, Amgen, Thermo Fisher Scientific, Eli Lilly, Diaceutics, GSK, Merck and AstraZeneca, Janssen, Diatech, Novartis and Hedera for work performed outside of the current study. Alessandro Russo reports personal fees from BMS, Novartis, Pfizer, AstraZeneca, MSD, Amgen and Roche for work performed outside of the current study. Given their roles in the journal, Umberto Malapelle (Editor-in-Chief), Francesco Pepe (Editorial Board Member), and Alessandro Russo (Editorial Board Member) had no involvement in the peer review of this article and have no access to information regarding its peer review. The other authors declare that they have no known competing financial interests or personal relationships that could have appeared to influence the work reported in this paper.
